# CemrgApp: An interactive medical imaging application with image processing, computer vision, and machine learning toolkits for cardiovascular research

**DOI:** 10.1016/j.softx.2020.100570

**Published:** 2020-07-31

**Authors:** Orod Razeghi, José Alonso Solís-Lemus, Angela W.C. Lee, Rashed Karim, Cesare Corrado, Caroline H. Roney, Adelaide de Vecchi, Steven A. Niederer

**Affiliations:** King’s College London, London, United Kingdom

**Keywords:** Image processing, Computer vision, Machine learning, Medical imaging, Interactive platform, Cardiovascular research

## Abstract

Personalised medicine is based on the principle that each body is unique and will respond to therapies differently. In cardiology, characterising patient specific cardiovascular properties would help in personalising care. One promising approach for characterising these properties relies on performing computational analysis of multimodal imaging data. An interactive cardiac imaging environment, which can seamlessly render, manipulate, derive calculations, and otherwise prototype research activities, is therefore sought-after.

We developed the Cardiac Electro-Mechanics Research Group Application (CemrgApp) as a platform with custom image processing and computer vision toolkits for applying statistical, machine learning and simulation approaches to study physiology, pathology, diagnosis and treatment of the cardiovascular system. CemrgApp provides an integrated environment, where cardiac data visualisation and workflow prototyping are presented through a common graphical user interface.

## 1 Motivation and significance

Precision cardiology requires an accurate characterisation of the disease phenotype for each patient [1]. Computational analysis on multi-modal imaging data is a promising approach for the characterisation of patient specific properties, which aids in the understanding of biological behaviour. Cardiovascular clinical researchers therefore require an environment, which can seamlessly render multiple time dependent images, enable manipulation of data in a flexible user defined workflow, display the results of derived calculations, and otherwise prototype their desired research activities.

Several research software packages have been developed to provide distinct functionalities with multimodal imaging data: 3D Slicer [2], Analyze (AnalyzeDirect, Overland Park, KS), Eidolon [3], and the Medical Imaging Interaction Toolkit (MITK) [4] are all amongst the examples. We developed Cardiac Electro-Mechanics Research Group Application (CemrgApp) as an MITK based platform extended with custom image processing and computer vision toolkits for applying statistical, machine learning and simulation approaches to study physiology, pathology, diagnosis and treatment of the cardiovascular system. CemrgApp provides an integrated environment, where cardiac data visualisation and workflow prototyping are presented through a common graphical user interface for high throughput clinical studies.

CemrgApp is specifically tailored to enable lean iterative developments, accelerate clinical translation, and reduce barriers to collaborative and interdisciplinary work. It increases the reusability of code by enabling clinical researchers to make use of earlier developments by computer vision scientists and perform advanced image analysis with limited training. CemrgApp has in fact provided a common platform for them to cooperate in various cardiac applications and preprocedural planning in clinical trials [5–10].

CemrgApp is easy to acquire, install, setup and use. It has been made publicly available as source code on Github^1^ and in binaries for Linux and macOS in version 1.0, as well as Microsoft Windows in version 2.0 of the application. To enhance modularity, Cem- rgApp features a dynamic Docker^2^ interface for facilitating the use of third party applications and integration with software from other centres. Building CemrgApp upon the more generic MITK software system prevents it from being *a one person project* and enables its users to engage with a bigger community for support.

## 2 Software description

The open-source MITK software system offers an infrastructure for construction of specifically tailored applications by combining Kitware platforms^3^ (the Insight Toolkit (ITK), the Visualisation Toolkit (VTK)) and other interactive medical imaging features. CemrgApp is built upon MITK and provides cardiovascular data analysis functionalities in the form of bespoke plugins, which are sub-programs with dedicated user interface environments. The current two major versions of CemrgApp v1.0 and v2.0 are compatible with MITK 2016.3.0 and MITK 2018.04.2, respectively.

**Fig. 1:**
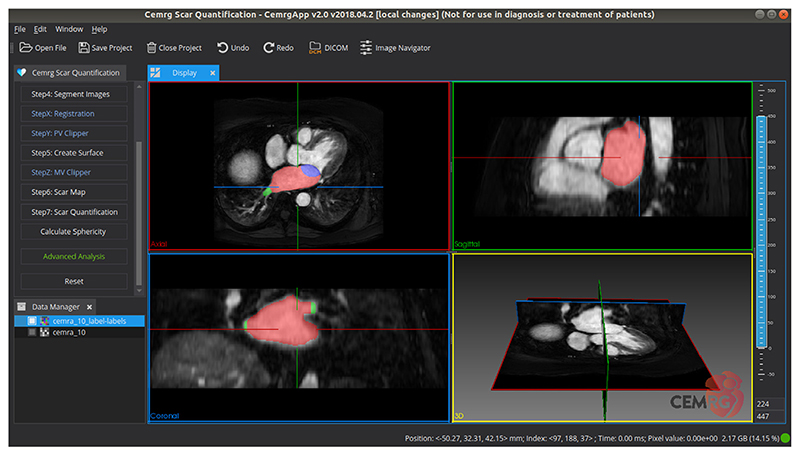
An example of CemrgApp user interface. The panel in the left corner displays a series of push buttons, sequentially numbered to present steps in a workflow. The panel in the bottom left corner is the data manager that allows the user to manipulate or visualise data. The 2D anatomical views and the 3D renderer window are presented under the display panel.

In CemrgApp, all plugins follow a similar and intuitive user interface that breaks complicated data manipulation pipelines into a series of sequential steps; each handled by a numbered button. A render window consisting of 2D and 3D views visualises data and dropdown menus modify the behaviour of the pipeline. An example of this interface can be seen in Fig. 1.

### 2.1 Software architecture

CermgApp is an object-oriented, cross-platform application implemented in C++. Similar to MITK, CemrgApp has been designed with the aim of providing a modular and reusable code base to enable rapid development of new features. Following this design philosophy, most CemrgApp classes are derived from top-level classes of MITK, which in turn are derived from ITK.

CemrgApp uses a data centred approach, similar to the *modelview-controller* design pattern. The *model* represents the application logic and controls the backend functions. Data objects and data trees are the central elements representing the model. The *view* displays the model data to the user, usually handled by the MITK core visualisation codes. Finally, the *controller* handles user input and forwards it to the model by calling the corresponding functions.

CemrgApp includes MITK default modules in addition to a dynamic Docker interface, facilitating the use of applications written in different programming languages with various dependencies. The Docker interface of CemrgApp relies on the Docker community edition and is licenced under the Apache license (version 2.0). CemrgApp at its current state uses the following packages in the form of Docker containers:

- The Computational Geometry Algorithms Library (CGAL^4^), which provides reliable geometric and meshing algorithms.
- The Medical Image Registration ToolKit (MIRTK^5^), which provides a collection of libraries and command line tools to assist in processing imaging data.
- Tensorflow [11], which is an end-to-end open source machine learning platform used for the computer vision algorithms.

### 2.2 Software functionalities

Software functionalities of the CemrgApp are organised as the following plugins:

#### 2.2.1 Motion quantification

**Fig. 2:**
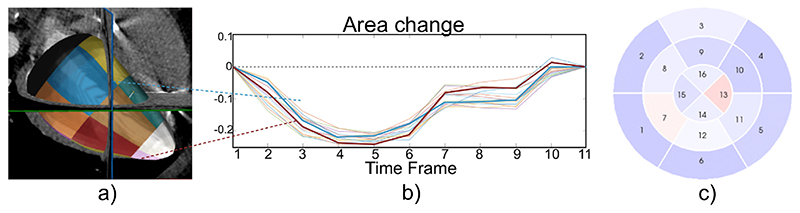
Analysing motion from cardiac CT is facilitated by: (a) an interactive renderer for visualising 3D images and surface meshes of the heart, (b) a plot over time frame illustrating deformation curves from 16 individual wall segments, and (c) a bullseye plot displaying a 16-segment map for visualisation of deformation at a specific time frame in the cardiac cycle, where larger deformations are illustrated in blue. (For interpretation of the references to colour in this figure legend, the reader is referred to the web version of this article.)

Recent radiation dose reduction techniques have made computed tomography (CT) scans more applicable and extracting heart function from cardiac images feasible. This plugin estimates cardiac motion by applying an image registration warping field to a triangulated mesh of the heart’s chambers. Fig. 2 displays an example use of this plugin for quantifying motion of the left ventricle chamber.

Tracking cardiac motion can be defined as the non-rigid registration of cardiac image sequences. In free-form deformation (FFD) registration [12], a non-rigid deformation ***h*** = [*X Y Z*]^*T*^ is represented using a B-spline model in which the deformation is parametrised using a set of control points ***Φ*** = [*D V W*]^*T*^. To be able to deal with large global deformations and to improve robustness, the classic FFD registration normally uses a multi-level representation [13].

The “Motion Quantification” plugin utilises an optimised temporal sparse free-form deformation (TSFFD) technique [14], which extends the classic FFD approach and recovers smoother displacement fields in the temporal domain by using a four-level representation and sum of squared differences as the similarity measure. The registration energy function is minimised using a gradient descent approach [15].

The cardiac motion can then be characterised by the circumferential and longitudinal strains as well as local area change throughout the cardiac cycle. The Green-Lagrange strain tensor **E** is calculated by $E=\frac{1}{2}\left(F^{T}F-I\right)$, where **I** is the identity tensor and **F** the deformation gradient tensor.

The “Anatomical Measurements” plugin can similarly perform a more generic motion estimation and track anatomical landmarks manually placed on the scans. It can then compute a number of measurements such as Euclidean distance between two landmarks as well as area and perimeter of a region of interest enclosed by the landmarks.

#### 2.2.2 Scar quantification

Atrial fibrillation (AF) is a heart condition that causes an irregular and often abnormally fast heart rate. Fibrosis is a major contributor to sustained AF. Late gadolinium enhancement (LGE) cardiac magnetic resonance imaging (CMR) is currently the only available tool for its non-invasive assessment. This plugin was developed to facilitate, visualise and validate the multiple analysis steps required for the assessment of fibrosis and quantification of scarred tissue.

**Fig. 3:**
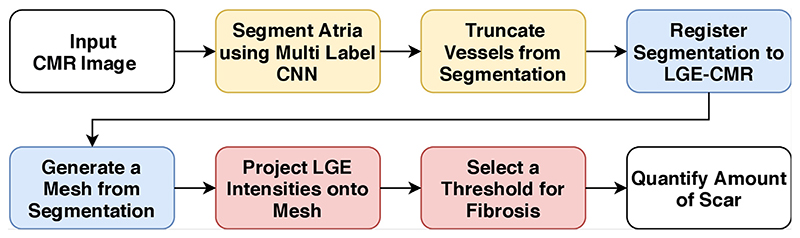
A fully automatic workflow for assessment of fibrosis and quantification of scarred tissue. A convolutional neural network (CNN) makes segmentation possible without user intervention. Deep learning components are in yellow. Blue represents conventional image processing techniques and red illustrates assessment of fibrosis using LGE-CMR. Quality control assessment is performed after every step in the workflow. (For interpretation of the references to colour in this figure legend, the reader is referred to the web version of this article.)

The plugin contains data processing toolkits, which perform resampling, automatic segmentation, rigid registration and transformation of images, bespoke smoothing of segmentations, LGE image interrogation, and assessment of fibrosis. All these steps are automated in an end-to-end workflow, as illustrated in Fig. 3.

**Fig. 4:**
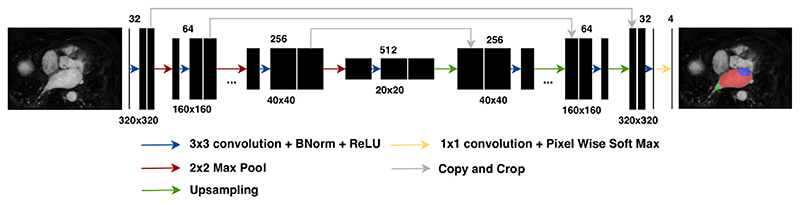
General network architecture with 5 concatenations between contracting and expanding paths. Network’s layers are illustrated with coloured arrows. (For interpretation of the references to colour in this figure legend, the reader is referred to the web version of this article.)

The workflow contains a multi-label convolutional neural network (CNN), designed to accurately delineate atrial structures including the blood pool, pulmonary veins and mitral valve. The output from the network removes the user dependent steps and allows for reproducible quantification of fibrosis from scans. The architecture of the network can be seen in Fig. 4.

The network was trained and tested on a dataset of 207 manually labelled scans and a 0.91 ±0.02 Dice score was achieved for atrial blood pool segmentation. The network was also checked against the “2018 Atrial Segmentation Challenge” dataset to evaluate its potential limitations on analysing different scans from a different centre. The network without any retraining on the challenge dataset achieved a Dice score of 0.80 ± 0.05. Retraining the network on this dataset achieved a Dice score of 0.89. Testing the network against the “2013 Left Atrial Segmentation Challenge” yielded a Dice score of 0.90 ± 0.09. Although our results show the robustness of the network when tested against these multi-centre datasets, there will be cases, where the network fails. In such cases, the user has the option to use the MITK manual segmentation tools and carry on with the rest of fibrosis quantification process.

The network was trained on 2D slices extracted from the 3D scans using a dedicated GPU machine. At the run time, the plugin initially slices the scan into 2D images, performs the prediction using the pretrained network, and finally puts the results back together. This method has been used in previous medical segmentation methods and helps with keeping the method computationally tractable without losing significant performance [16, 17].

**Fig. 5:**
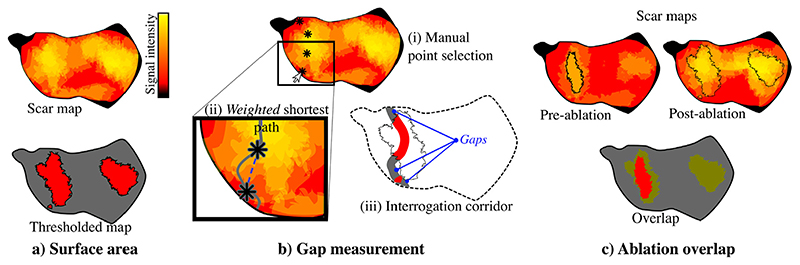
Advanced Scar Calculations Diagram. (a) The scar tissue is identified from triangular elements of the surface mesh, which have a fibrotic score above a given threshold. (b) A semi-automatic method allows the creation of an exploration corridor around the veins to measure ablation lesion gaps, which are areas in the corridor where scar values are below the threshold. (c) The ablation overlap comparison is performed by comparing the scar tissue in both pre- and post-procedure maps.

Additionally, the “Advanced Calculations” toolkit of this plugin can quantify extra features in the scar tissue, with an emphasis on the tissue’s status before and after pulmonary vein isolation (PVI). PVI is a common ablation procedure that prevents abnormal electrical signals from activating the atrium by electrically isolating the pulmonary veins. A successful ablation produces a lesion encircling the veins that stops the activation [18]. Fig. 5 shows the toolkit’s main functions: (a) measure the surface area of the scar tissue, (b) measure the number of gaps around a vein and (c) compare the pre- and post-ablation scars.

#### 2.2.3 Morphological measurements

Remodelling of heart chambers is a common feature of many cardiovascular conditions and is a sensitive marker of adverse cardiovascular outcomes. The aim of this plugin is to analyse remodelling of heart’s chambers from cardiac scans by assessing volume, surface area, wall thickness, and associated vessels morphological characteristics.

The plugin provides a semi-automatic method of segmenting the blood pool and the cardiac wall using an iterative growing algorithm, which detects pixels within a signal intensity range corresponding to muscular tissue of the heart. A high-resolution tetrahedral mesh is then constructed from the wall segmentation using CGAL. The mesh is subsequently processed to tag the endocardial (inner) and epicardial (outer) surface layers.

To calculate tissue thickness, the Laplace equation (Δ^2^ *u* = 0) is solved with Dirichlet boundary conditions assigned at the endocardial *u* = 0 and epicardial *u* = 1 surfaces to generate a series of nested iso-potential surfaces. Wall thickness is evaluated as the length of the path between the endocardium and epicardium when moving orthogonally between adjacent isopotential surfaces. Tissue thickness measurements are associated with each mesh node.

**Fig. 6:**
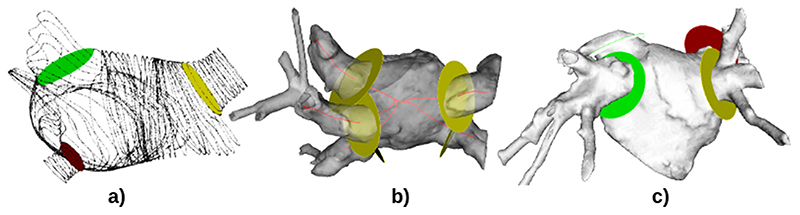
(a) A Voronoi diagram displaying partitioning of the geometry into polygons with area of surrounding structure encoded. (b) Computed centrelines are illustrated in red. The localisation of the opening points is achieved by analysing the change in the area of the surrounding structure. (c) The colourful disks represent different clipping techniques. (For interpretation of the references to colour in this figure legend, the reader is referred to the web version of this article.)

In order to truncate vessels and calculate their morphological characteristics, a Voronoi diagram [19] is utilised by the plugin. This diagram is extracted from a surface mesh made from the blood pool segmentation, as seen in Fig. 6(a). Each of the vessels is initially identified by the user placing landmarks on the distal ends of the mesh in a 3D VTK renderer, which was specifically designed for dealing with user interactions more effectively than the default MITK 3D visualisation window. Centrelines (Fig. 6(b)) are then automatically drawn from these points to the centre of the body using the VMTK library. As the centrelines enter the body, the maximum area of the surrounding structure increases significantly. This inflection is used to identify the opening of the chamber [20]. Then, a fully automatic clipper computes the geometric properties of the vessels’ inner walls and truncates the blood pool precisely at the opening point.

In addition to the fully automatic clipper (yellow disk in Fig. 6(c)), the plugin also provides two other types of truncation methods with different levels of manual interventions to provide flexibility. An example of the fully manual method is shown as a red disk, where the user picks a number of seeds on the mesh to define a contour. These seeds generate a custom shaped plane, which is then used for truncation of the vessels. The green disk is the semi-automatic method, in which the user set the size of clipping plane.

## 3 Illustrative examples

The process of assessing fibrosis manually is described in this section. By selecting the “Scar Quantification” plugin, the user is presented with the following steps:

1. The first step loads and visualises CMR images. Then, the step of processing images converts the scans from the DICOM^6^ format into anonymised NIfTI^7^ format.
2. The segmentation step can be done either automatically by the convolutional neural network or semi-automatically by utilising a region growing tool to delineate the atrium.
3. Alignment of the segmentation with the LGE scan is performed using the MIRTK rigid registration option [21].
4. To isolate the LA cavity from the segmentation, Cemr-gApp uses the automatic localisation and vessel truncation tool. A manual slider is also available to reposition the truncation disks more proximal or distal, if needed.
5. In the next step, a surface mesh is created from the truncated segmentation. The mitral valve can also be clipped from the mesh by manipulating the visualisation renderer.
6. Normals are taken, 3 mm externally and 1 mm internally, to the nodes of the mesh and a maximum intensity projection technique is used to interrogate the LGE scan and identify fibrotic regions.
7. Finally, global scar burdens are calculated using predefined thresholds.

An explanatory video of this example is available as a supplement.

## 4 Impact

By tailoring CemrgApp to the clinical researchers’ needs and technical abilities, we aimed to accelerate clinical translation and allow users to produce automated and reproducible results, which reduce intra and interobserver variability in the studies. By reducing variability, the number of patients required to answer a specific clinical question is reduced. This allows smaller, less expensive and faster clinical trials to be performed. Likewise, the ability to process large datasets with standardised imaging structures permits users to create virtual cohorts of digital twins at new significant scales.

To date, CemrgApp has been used by clinical researchers for: improved co-registration of ex-vivo and in-vivo CMR images [5], reproducibility assessment of atrial fibrosis quantification [6], evaluation of left atrial scar formation using an ablation index- guided point-by-point workflow [7], optimisation of LGE-CMR imaging of post ablation atrial scar in a cross-over study [8], quantification of mitral valve geometry on multi-slice CT [9], and guidance of lead placement in CRT [10].

All these successful studies provided cross user providence for the analysis of each case and gave confidence that the produced results were not user dependent. CemrgApp has also provided commercial services as part of the Medtronic’s Fire and Ice II trial (Clinical Trials Gov Identifier: NCT03706677), which is the pilot phase of a prospective, randomised, single-blinded, multi-centre, interventional clinical trial for comparing efficacy and safety of isolation of the PVI using catheters in subjects with persistent AF.

Distribution of research software developed in academia is challenging. The process of delivering a newly discovered algorithm, a novel computational model, or even a simple batch script to its potential end users is not straightforward. Distribution by source is an option but expecting the end user to deploy the right compiler, the correct version of third party libraries, and the compatible system architecture is too optimistic, if not unreasonable. Our selected method of deployment addressed these challenges by making self-contained standalone executables available for each operating system. Furthermore, utilising Docker allowed the application to be lightweight enough to run on clinicians’ laptops, instead of dedicated onsite machines.

## 5 Conclusions

Developing novel but complicated medical imaging workflows, which are not user friendly, verifiable, and reproducible across multiple centres, can hinder the process of translation into clinic. Interactive features of CemrgApp allow visualisation and manipulation of cardiovascular data in an easy reproducible environment for end users to explore innovative ideas and pave the way for future clinical research.

## Code metadata

**Table:**

Current code version	2.0
Permanent link to repository used for this code version	https://github.com/ElsevierSoftwareX/SOFTX_2020_173
Legal code license	BSD 3-Clause License
Code versioning system used	Git
Software code languages, tools, and services used	C++
Compilation requirements, operating environments & dependencies	Minimum Required: CMake 3.14.5, Qt 5.12, VMTK 1.4, and GCC version 5.4.0 or Apple LLVM 10.0 or Visual Studio 2017 version 15.9
Link to developer documentation/manual	http://www.cemrgapp.com
Support email for questions	info@cemrgapp.com

## Software metadata

**Table:**

Current software version	2.0
Permanent link to executables of this version	http://www.cemrgapp.com
Legal software license	BSD 3-Clause License
Computing platforms/operating systems	Linux, macOS, Microsoft Windows
Installation requirements & dependencies	Docker 18.03.1-ce for advanced toolkits
Link to user manual	http://www.cemrgapp.com
Support email for questions	info@cemrgapp.com

## Footnotes

1
https://github.com/

2
https://www.docker.com

3
https://www.kitware.com

4
https://www.cgal.org

5
https://mirtk.github.io

6
http://medical.nema.org

7
http://nifti.nimh.nih.gov
